# Closing the Gaps: Testing the Efficacy of Carbapenem and Cephalosporin Treatments of Late-Stage Anthrax in Rabbits

**DOI:** 10.3390/pathogens13110936

**Published:** 2024-10-28

**Authors:** Assa Sittner, Elad Bar-David, Itai Glinert, Amir Ben-Shmuel, Josef Schlomovitz, Haim Levy, Shay Weiss

**Affiliations:** Department of Infectious Diseases, Israel Institute for Biological Research, Ness-Ziona P.O. Box 19, Israel; assas@iibr.gov.il (A.S.); eladb@iibr.gov.il (E.B.-D.); itaig@iibr.gov.il (I.G.); amirb@iibr.gov.il (A.B.-S.); yosi.shlo@gmail.com (J.S.)

**Keywords:** *Bacillus anthracis*, anthrax, doxycycline, meropenem, cefazolin, CDC guideline: CNS infection, antibiotic treatment

## Abstract

Anthrax is a fatal zoonotic disease caused by exposure to *Bacillus anthracis* spores. The CDC’s guidelines divide anthrax treatment into three categories according to disease progression: post-exposure prophylaxis (PEP), systemic, and systemic with a suspicion of CNS infection. While the prognosis for PEP or the early treatment of systemic anthrax is very good, ingress of the bacteria into the CNS poses a substantial clinical challenge. Here, we use rabbits to test the efficacy of a combined treatment of meropenem and doxycycline, which is the first choice in the CDC recommendations for treating systemic patients with an indication of CNS infection. In addition, we test the efficacy of the first-generation cephalosporin, cefazolin, in treating different stages of the disease. We found that the combination of doxycycline and meropenem is highly effective in treating rabbits in our inhalation model. Cefazolin was efficient only for PEP or systemic-stage treatment and not for CNS-infected animals. Our findings support the CDC recommendation of using a combination of doxycycline and meropenem for systemic patients with or without indications of CNS infection. We found that cefazolin is a decent choice for PEP or early-stage systemic disease but recommend considering using this antibiotic only if all other options are not available.

## 1. Introduction

Anthrax is a zoonotic disease that mainly, but not solely, affects grazing animals [1,2]. Its natural transmission to humans is rare and usually results from close contact with sick animals or contaminated animal products [3,4]. Anthrax is caused by the spore-forming, Gram-positive bacteria *Bacillus anthracis*. In most cases, the spore is the infectious form. Inside the host, the spore germinates and, in response to specific stimuli, produces two main virulence factors: tripartite toxins and a capsule, encoded by the two virulence plasmids pXO1 and pXO2 [1,2]. The toxins inactivate the host’s immune system, and the capsule protects the bacteria inside phagocytic cells and also has an immune modulation effect. The tripartite toxin is composed of the lethal factor (LF), an endopeptidase specific for proteins of the MAP kinase pathway; the edema factor (EF), a calmodulin-dependent adenylate cyclase that disrupts cell cycle regulation; and protective antigen (PA), which binds to a specific receptor present on all cell types, forming a heptamer that transports the LF and EF into the cytoplasm. The *B. anthracis* capsule is a homo-polymer of γ-D-glutamic acid that forms a negative charged helix, creating a barrier surrounding the bacterium [5,6].

The type of disease seen in humans is determined by the route of infection: contact with skin lesions, injection, digestion, or inhalation. The contact of spores or vegetative bacteria with compromised skin results in the most obvious and typical type of anthrax—cutaneous [1]. This form of the disease manifests as the painless formation of a typical lesion (eschar) surrounded by an inflamed region, which in most cases heals with minimal scarring, even in the absence of antibiotic treatment. Fatality from this form of infection is between 5 and 30% and is the result of the transition of the infection from a localized to systemic mode [7]. The resemblance of eschar to coal is the name source of the disease and bacterium (ἄνθραξ, anthrax in Greek) [3]. The second form of infection, which is becoming the most common, is due to the digestion of contaminated meat from a near-death animal, domestic or wild, resulting in gastrointestinal disease [8]. This form of the disease is usually 100% lethal in the absence of antibiotic treatment and starts as a severe gastric inflammation and rapidly becomes systemic. Another form of this disease is oropharyngeal, where the infection causes a swelling of the lymph nodes in the throat that leads to suffocation [9]. Since the accepted paradigm is that sporulation occurs postmortem as the contaminated blood is exposed to air [4], it is assumed that the vegetative form of the bacterium is responsible for this form of the disease. Deep-tissue anthrax resulting from injecting spore-contaminated heroin was reported in the early 2010s in Europe [10]. This massive tissue inflammation progressed, in the absence of antibiotic treatment, into systemic infection and death [11]. Unlike the cutaneous and oropharyngeal diseases, there have been no new reports of the deep-tissue form of the disease in the last decade. Though not considered a natural disease [12,13,14], inhalation anthrax is the most worrying form of the disease, which is where *B. anthracis* spores are used as a military or bio-terror weapon [15,16,17]. The inhalation of spores results in a systemic disease without any early typical symptoms other than those resembling the common flu [1]. The acute stage of the disease is short and lethal even with antibiotic treatment [18]. Inhalation anthrax was considered an occupational disease that affected workers in goat-hair-processing mills [13]. Two major deadly events demonstrated the potential of using *B. anthracis* spores as a weapon: the Sverdlovsk accident [19,20,21] and the 2001 anthrax letter attacks [22,23]. In Sverdlovsk, accidental spore discharge caused the death of people and farm animals up to 50 km from the source. In 2001, spores that leaked from sealed envelopes caused pulmonary anthrax in 11 people, several of them without any traced contact with the identified spore-containing articles [18,23]. The systemic dissemination of the bacteria from the initial infection into the bloodstream is common to all forms of the disease. From the bloodstream, the bacteria invade the internal organs and eventually cross the blood-brain barrier (BBB), causing meningitis and hemorrhage, which are most likely fatal [1,2,20,24,25,26,27].

The treatment of anthrax patients is mainly based on antibiotics [28]. Currently, the CDC treatment recommendations divide the treatment into post-exposure prophylaxis (PEP) and the treatment of systemic (symptomatic) disease, depending on the stage of the disease [28]. The treatment of exposed, non-symptomatic individuals (PEP) relies on the oral administration of a single antibiotic, usually ciprofloxacin or doxycycline. The treatment of symptomatic patients is more complicated, and treatment with more than one antibiotic is recommended due to the possibility of meningitis, which is common in the late stages of the disease in humans and animal models. Since data from actual patients are limited due to poor documentation and the low number of cases in the Western world, most of the data concerning treatment efficacy are based on experiments in animal models (mainly NHP and rabbits). Previously, we and others demonstrated the efficacy of antibiotic treatment at different stages of the disease, from PEP through symptomatic animals to CNS-infected animals [29,30,31,32,33,34,35]. In its 2023 guidelines, the CDC’s first choice for the treatment of symptomatic adults is the combination of meropenem and doxycycline (minocycline) [28]. While we tested meropenem as a single treatment in rabbits [30,36], the combined treatment was not tested. In addition, though cephalosporins are not recommended for anthrax treatment, the CDC acknowledged the lack of experimental information regarding the efficacy of cefazolin, a first-generation cephalosporin, in treating anthrax [28]. Previously, we demonstrated in guinea pigs that PEP with cefazolin resulted in full protection in the first 3 days, followed by the subsequent death of 50% of the infected animals while under antibiotic treatment [29]. We propose that, although sensitive in vitro, *B. anthracis* expresses β-lactamases that enable the bacteria to grow despite this antibiotic treatment. Herein, we test the efficacy of cefazolin treatment in rabbits at different stages of the disease, including PEP, different stages of systemic disease, and with CNS infections. In addition, we test the efficacy of the combined treatment of high-dose meropenem and doxycycline in treating rabbits at different stages of systemic disease following airway infection.

## 2. Materials and Methods

### 2.1. Bacterial Strains, Media, and Growth Conditions

*B. anthracis* Vollum strain (ATCC14578) [37] was used in this study. Spores were used for IN instillation or nasal sprays. For CNS infections, *B. anthracis* spores were germinated by incubation in Terrific broth (Merck T9197) for 30 min, followed by 2 h of incubation in DMEM-10% NRS at 37 °C in a 10% CO_2_ atmosphere to induce capsule formation.

### 2.2. Infection of Rabbits

New Zealand white rabbits (2.5–3.5 kg) were obtained from Charles River (Saint Constant, Canada). The animals received food and water ad libitum.

Intranasal instillation (PEP). Rabbits were anesthetized using 100 mg of ketamine and 10 mg of xylazine and infected by pipetting small drops of spore suspension into their nasal cavities with a 1 mL tip. The infection dose was 1 × 10^6^ CFU of spores (10xLD_100_) in a total volume of 1 mL. Animals were treated with antibiotics 6–24 h post-inoculation (as described in the Results).

Airway infection. Rabbits were anesthetized using 100 mg of ketamine and 10 mg of xylazine and infected by spraying a spore suspension into their nasal cavities with a MicroSprayer^®^ Aerosolizer for Rat—Model IA-1B-R (PennCentury™ Wyndmoor, PA USA). The infection dose was 1 × 10^4^ CFU of spores (10xLD_100_). Following infection, the instrument was visually inspected for signs of blood, which might imply nasal cavity injury and direct bloodstream spore deposition. In all cases, the device was confirmed negative. Blood samples were drawn from the rabbits’ ear veins to determine bacteremia by total viable counts 16 h post-inoculation (CFU.mL^−1^), and the animals were immediately treated with antibiotics (as described in the Results).

CNS infection. For ICM administration, the animals were anesthetized using 100 mg of ketamine and 10 mg of xylazine. Using a 23 G blood collection set, 300 μL of encapsulated vegetative bacteria were injected into the cisterna magna. The remaining sample was plated for total viable counts (CFU.mL^−1^). The animals were observed daily for 14 days or for the indicated period.

### 2.3. Antibiotic Treatment

Following infection, animals were treated as described in the results. Antibiotic doses were as follows: doxycycline 15 mg/kg (doxycycline—ratiopharm SF 100 mg/5 mL), meropenem 40 or 150 mg/kg (Anfarm Hellas S.A. Kifisia Greece, 500 mg I.V.), cefazolin 30 or 60 mg/kg (cefazolin, Panpharma S.A. Luitre France, 1 g). The MIC for the Vollum strain; doxycycline is 0.016–0.032 μg/mL (Etest), meropenem is 0.064 μg/mL (by Etest) and cefazolin is 0.15 μg/mL (microdilution).

### 2.4. Ethics

This study was carried out by trained personnel in strict accordance with the recommendations of the *Guide for the Care and Use of Laboratory Animals* of the National Research Council. All protocols were approved by the IIBR committee on the Ethics of Animal Experiments (RB-14-18, RB-02-20). We used female rabbits in these experiments since there are no significant differences in *B. anthracis* pathogenicity between male and female rabbits [38]. Before inoculation, animals were sedated using 100 mg of ketamine and 10 mg of xylazine. The animals were monitored twice a day and euthanized immediately with a 120 mg/kg sodium pentobarbitone injection when one of the following symptoms was detected: severe respiratory distress or the loss of righting reflex. Animals unable or unwilling to drink were injected with 20–100 mL of saline or dextrose isotonic solution SC. Since, in most cases, anthrax symptoms are visible only in close proximity to death, there were cases where animals succumbed to the disease.

### 2.5. Statistical Analysis

The significance of the differences in survival rates between treated groups and untreated controls, as well as the differences in bacteremia and time to death, were determined by Log-rank, using Prism 6 software (GraphPad, Boston, MA, USA). For each cefazolin treatment experiment, the minimal group size was based on past experiments (n = 4 or 8). For the combined treatment of meropenem and doxycycline, we used an optimal group size comparable to our previous experiments [31].

## 3. Results

### 3.1. The Efficacy of Cefazolin as Post-Exposure Prophylaxis

**Figure 1:**
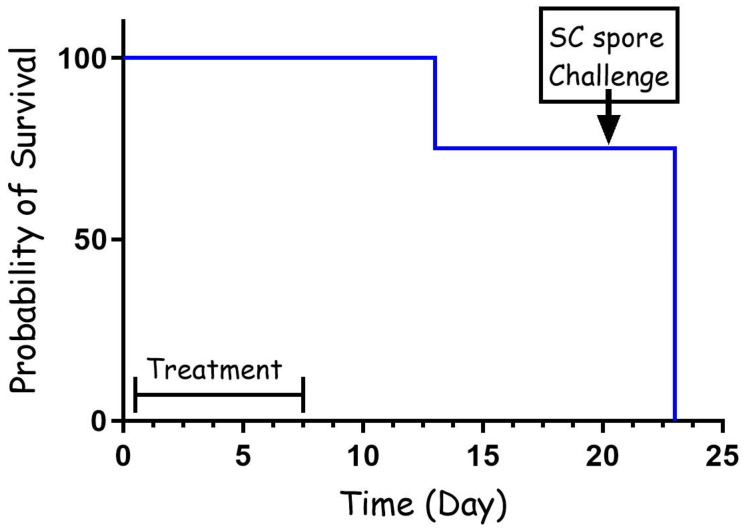
PEP treatment (60 mg/kg) with cefazolin following IN spore instillation of B. anthracis Vollum strain. Four rabbits were sedated and infected IN with spores. Treatment was initiated 6 h post-infection, SC, with 60 mg/kg cefazolin, twice a day. The treatment duration is marked by a vertical arrow and the SC spore challenge is marked by a horizontal arrow. Survival is presented as a Kaplan–Meier curve.

To test the effectiveness of cefazolin as PEP, rabbits were infected with virulent B. anthracis Vollum spores via intranasal instillation (IN) and treated intravenously (IV) 6 h post-infection with 60 mg/kg of cefazolin. The animals were then treated twice a day subcutaneously (SC) with the same dose for a total of 7 days. The animals were monitored for 14 days from the end of antibiotic treatment for signs of illness or death. Twenty-one days from infection the animals were challenged SC with 100 LD50 of Vollum spores to test for any protective immune response that developed during the antibiotic treatment. The results of the experiment are presented in Figure 1. The results show that all the animals (n = 4) survived for 12 days, while one rabbit succumbed to the infection 6 days from the end of the antibiotic treatment. The other three rabbits survived for 21 days. However, none of them developed protective immunity, and they all succumbed to the SC challenge.

**Figure 2:**
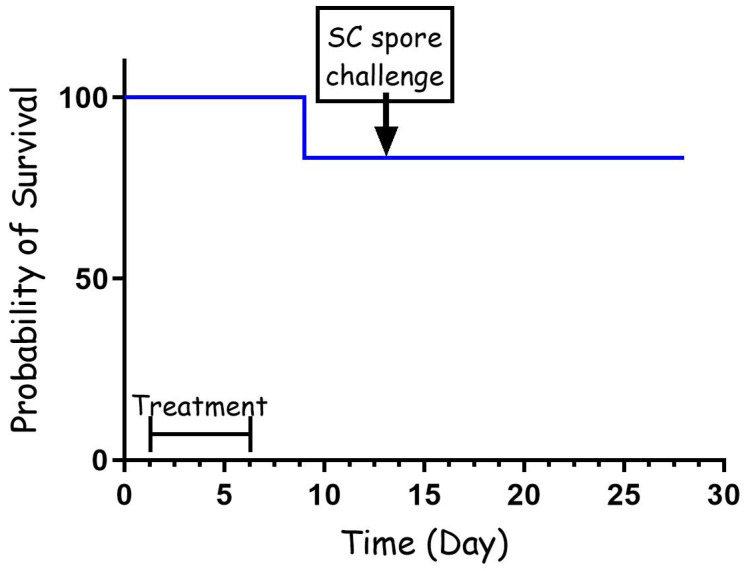
PEP treatment (30 mg/kg) with cefazolin following IN spore instillation of B. anthracis Vollum strain. Eight rabbits were sedated and infected IN with spores. Treatment was initiated 24 h post-infection, SC, with 30 mg/kg of cefazolin, twice a day. The treatment duration is marked by a vertical arrow, and the SC spore challenge is marked with a horizontal arrow. Survival is presented as a Kaplan–Meier curve.

To further test the effectiveness of the cefazolin treatment, we repeated the PEP experiment with three major modifications. For this experiment, the antibiotic dose was reduced from 60 mg/kg to 30 mg/kg, the first treatment was administrated 24 h from the IN spore instillation, and the treatment lasted for a total of 5 days. The results presented in Figure 2 demonstrate that the short, low-dose treatment was as effective as the full-dose experiment. In this experiment, one rabbit succumbed to the infection, while seven rabbits were protected for 14 days from the IN infection. Furthermore, all the animals that survived post-antibiotic treatment developed protective immunity that protected them from a lethal spore challenge of 100 LD50, having survived for 14 additional days, after which they were euthanized.

### 3.2. The Efficacy of Cefazolin as a Treatment for Systemic Disease

**Figure 3:**
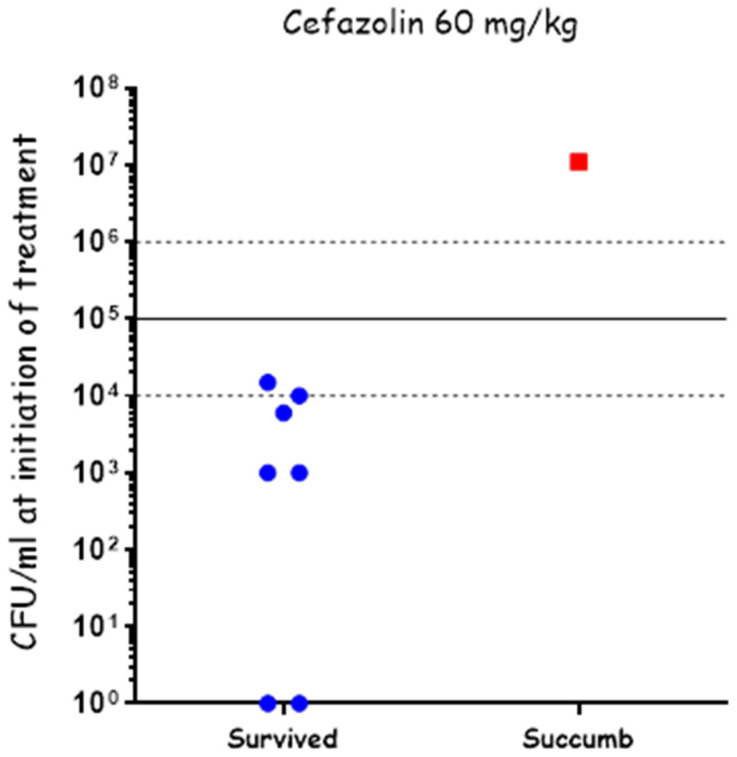
Cefazolin treatment of systemic disease following IN spray of *B. anthracis* Vollum spores. Rabbits were inoculated by spraying spores into their nasal cavity using a MicroSprayer^®^ Aerosolizer. Blood bacterial concentrations were determined at the time of treatment initiation. Survival (blue) or death (red) is presented in correlation to initial bacteremia. The disease stage is indicated by bacteremia levels: 10–10^4^ CFU/mL—moderate, 10^4^–10^5^ CFU/mL—Severe, >10^5^ CFU/mL—acute.

To test the effectiveness of cefazolin in treating systemic disease, we infected the rabbits IN using our PennCentury device to spray Vollum spores intranasally, as described previously. 20 h post-infection, a blood sample was collected from each of the rabbits to determine their bacteremia level (CFU/mL), after which they were treated IV with 60 mg/kg of cefazolin. The bacteremia level was determined by serial dilutions and plating, with the actual counting performed the next day. At treatment initiation, two rabbits were below the limit of detection (10 CFU/mL), five rabbits had bacteremia of 1 × 10^3^ to 2 × 10^4^ CFU/mL, and one rabbit had bacteremia of 1 × 10^7^ CFU/mL (Figure 3). The rabbits were treated SC, twice a day, for a total of 10 days with 60 mg/mL of cefazolin and monitored for 21 days post-infection. Cefazolin was effective in treating all the rabbits with bacteremia of up to 2 × 10^4^ CFU/mL and failed in treating the rabbit with 1 × 10^7^ CFU/mL (Figure 3). These results indicate that cefazolin is effective in treating rabbits with anthrax as PEP and in systemic disease of up to at least 2 × 10^4^ CFU/mL.

### 3.3. The Efficacy of Cefazolin as a Treatment for the Meningitis Stage of the Disease

**Figure 4:**
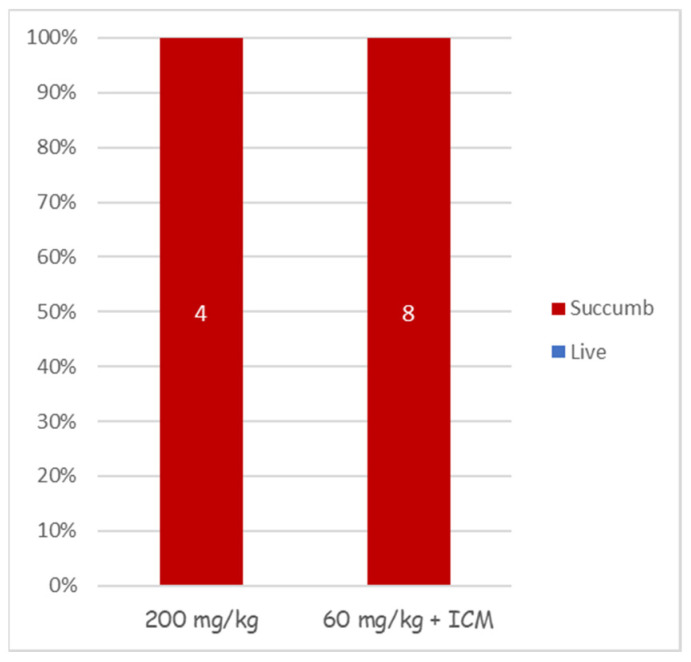
Cefazolin treatment efficacy in CNS-infected rabbits. Groups of 4 or 8 rabbits were infected by an injection of capsular vegetative Vollum bacteria into their cisterna magna and treated with cefazolin as indicated. Their survival (blue) and succumbed (red) percentages are presented.

Treating anthrax meningitis is challenging. Previously, we demonstrated that antibiotic treatment could protect rabbits from lethal CNS infections [30,33,36]. To determine the efficacy of cefazolin in treating anthrax-related meningitis, we used our rabbit CNS infections model [36]. Rabbits were injected with 3 × 10^4^ CFU of vegetative encapsulated bacteria of the fully virulent Vollum strain. Six hours post-infection, antibiotic treatment was initiated. To ensure maximal efficacy, we tested a regimen of a first dose of 200 mg/kg antibiotic administered IV, or a combination of 60 mg/kg IV with 300 μL of 20 mg/mL administered ICM, directly into the CSF. In both cases, the first treatment was followed by SC 60 mg/kg doses twice a day. None of the treatments were effective (Figure 4), as all of the infected animals died within 24 h of the infection, indicating that cefazolin is not effective in treating the meningitis stage of the disease.

### 3.4. The Efficacy of the CDC-Recommended Treatment of Systemic Patient Combining Meropenem and Doxycycline

**Figure 5:**
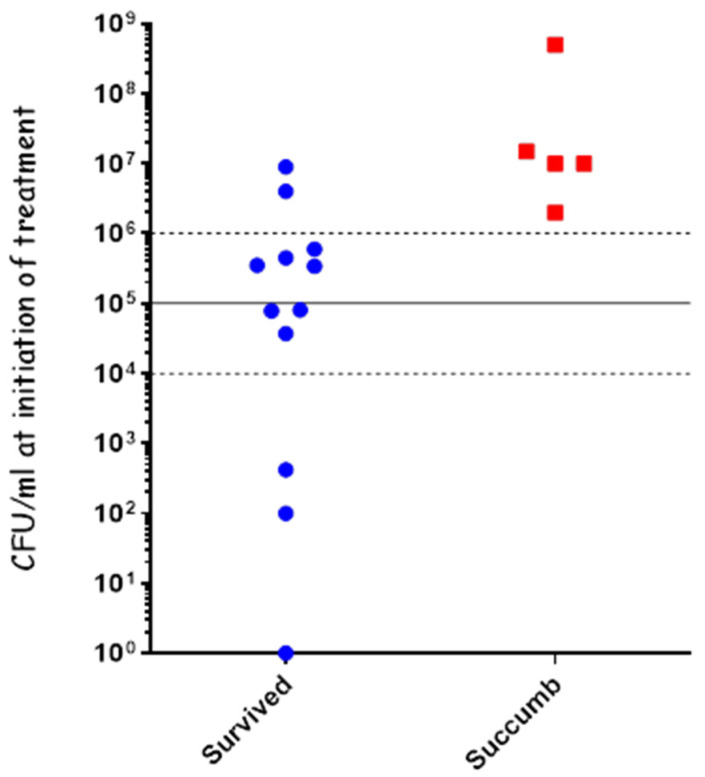
Combined treatment with meropenem and doxycycline of systemic disease following IN spray of B. anthracis Vollum spores. Rabbits were inoculated by spraying spores into the nasal cavity using a MicroSprayer^®^ Aerosolizer. Blood bacterial concentrations were determined at the time of treatment initiation. Survival (blue) or death (red) is presented in correlation to the initial bacteremia. Disease stage is indicated by bacteremia levels: 10–10^4^ CFU/mL—moderate, 10^4^–10^5^ CFU/mL—severe, >10^5^ CFU/mL—acute.

The recent CDC guidelines for the treatment of adults with confirmed anthrax recommends a combined treatment of meropenem and doxycycline [28]. Previously, we demonstrated that treatment with either meropenem (imipenem) or doxycycline as a monotherapy was effective in treating different stages of systemic anthrax [31,33]. Since imipenem is not recommended for treating CNS infections in humans due to its risk of inducing seizures [39], we previously tested meropenem in our rabbit CNS model. Our findings indicated that only a high-dose treatment of meropenem (150 mg/kg) was effective in treating anthrax meningitis in the rabbit CNS infection model [30,36]. Herein, we tested the efficacy of a combined administration of high-dose meropenem (150 mg/kg) and doxycycline (15 mg/kg) to treat inhalation anthrax at different stages of the disease. Rabbits (n = 17) were inoculated using the PennCentury device to spray Vollum spores as described previously [33]. 20 h post-infection, a blood sample was collected from each of the rabbits to determine their bacteremia level (CFU/mL), followed by an IV treatment of 150 mg/kg of meropenem and 15 mg/mL of doxycycline. The variation in bacteremia was high, and we treated animals with bacteremia levels in the wide range of <10–5 × 10^8^ CFU/mL, representing early-to-advanced stages of the disease (Figure 5). The combined treatment was highly effective, protecting all the animals with bacteremia of up to 1 × 10^6^ CFU/mL (n = 10); 33% of the animals with bacteremia of 1 × 10^6^ to 1 × 10^7^ CFU/mL; and failing to protect the single animal with bacteremia of 5 × 10^8^ (Figure 5). Overall, this combined treatment was as affective as treating with doxycycline as a monotreatment, indicating that there is no antagonism between these two drugs.

## 4. Discussion

Cephalosporins play a crucial role in the ICU setting, primarily due to their broad-spectrum antibacterial activity and relatively low toxicity [40]. Additionally, there are five generations of this drug that are specific for different types of bacteria [41]. During the anthrax letter attacks, there were several cases in which patients were misdiagnosed and treated with cephalosporins, leading to the deterioration of their condition and resulting in several deaths [18]. Generation 1 is the most relevant to Gram-positive bacteria. *B. anthracis* is sensitive to this drug in vitro [29,42]. In this manuscript, we tested, in a rabbit model, the efficacy of cefazolin, a generation 1 cephalosporin, in treating anthrax at different stages of the disease. As post-exposure prophylaxis, we tested relatively short treatments of 5 to 7 days administrated twice a day with 60 mg/kg or 30 mg/kg. The efficacy of these treatments was relatively high, protecting 85% of the animals (10/12) in both experiments (Figure 1 and Figure 2). The 60 mg/kg treatment, starting 6 h post-infection, was so effective that none of the surviving animals developed protective immunity, indicating that in these animals the spores did not have the opportunity to establish an infection (Figure 1). On the other hand, the short treatment was not always effective, allowing relapse and the development of lethal disease as soon as 4 days after the end of the antibiotic treatment. A longer antibiotic treatment, such as the recommended 30 to 60 days, will probably prevent this relapse. Challenging this treatment by initiating the antibiotic treatment 24 h post-infection and using 50% of the dose (30 mg/kg) for 5 days resulted in similar results (Figure 2). In this case, seven rabbits survived the treatment and the monitoring period, while one rabbit succumbed to the infection two days after treatment termination. However, in this case, all the surviving rabbits developed protective immunity, surviving a SC challenge 14 days post-infection. This protective immunity indicates that the spores germinated, secreted toxins, and established an infection, enabling the host to initiate a specific immune response. This could be a result of the late treatment initiation, low antibiotic dose, or both.

As for treating symptomatic animals, cefazolin was effective in treating rabbits with bacteremia of up to 10^4^ CFU/mL (experiment limitation). All attempts to use cefazolin for the treatment of meningitis failed, including a very high dose of 200 mg/mL and boosting the treatment with a direct injection of the antibiotic into the CSF, bypassing the need for crossing the BBB.

## 5. Conclusions

Altogether, according to the rabbit model, cefazolin can serve as an alternative for second-line antibiotics in cases of resistance, but only as a PEP or for early-stage patients. The combination of meropenem and doxycycline, the CDC’s first-choice treatment for systemic anthrax patients is highly effective, at least according to the rabbit pulmonary model [31,36]. Since we have already established that doxycycline is effective as a monotherapy under these conditions [32], the most important finding is that these substances do not inhibit each other. According to our previous findings, meropenem was effective in treating CNS infections only in high doses [30,31]. However, the dosage in the CDC guidelines was derived from experience in treating meningitis caused by different bacteria [28] and would likely be sufficient for *B. anthracis*, especially since the treatment includes a tetracycline (minocycline or doxycycline) with proven efficacy [32,33].

## Data Availability

All the data are presented in this manuscript.
